# Diagnostic performance of quantitative measures from [^18^F]FDG PET/CT, [^18^F]FEC PET/CT, and DW-MRI in the detection of lymph node metastases in endometrial and cervical cancer: data from the MAPPING study

**DOI:** 10.1007/s00259-025-07587-3

**Published:** 2025-11-03

**Authors:** Ben G King, Nishat Bharwani, William Wilson, Gary J.R Cook, Aslam Sohaib, Marielle Nobbenhuis, Victoria Warbey, Marc E. Miquel, Dow-Mu Koh, Katja N De Paepe, Pierre Martin-Hirsch, Sadaf Ghaem-Maghami, Christina Fotopoulou, Helen Stringfellow, Sudha Sundar, Ranjit Manchanda, Anju Sahdev, Allan Hackshaw, Tara D. Barwick, Andrea G. Rockall

**Affiliations:** 1 The Institute of Genetics & Cancer, The University of Edinburgh, Edinburgh, UK; 2 Department of Radiology, Imperial College Healthcare NHS Trust, London, UK; 3 Department of Surgery and Cancer, Faculty of Medicine, Imperial College London, London, UK; 4 Cancer Research UK & UCL Cancer Trials Centre, London, UK; 5 King’s College London and Guy’s and St Thomas’ PET Centre, School of Biomedical Engineering and Imaging Sciences, King’s College London, St. Thomas’ Hospital, Westminster Bridge Road, London, UK; 6 Department of Radiology, Royal Marsden Hospital NHS Foundation Trust, London, UK; 7 Department of Gynaeoncology, Royal Marsden Hospital NHS Foundation Trust, London, UK; 8 King’s College London and Guy’s and St Thomas’ PET Centre, Guy’s and St Thomas’ NHS Foundation Trust, London, UK; 9 NIR & MR Physics, Department of Clinical Imaging and Medical Physics, Guys and St Thomas’s NHS Foundation Trust, London, UK; 10 School of Biomedical Engineering and Imaging Sciences, King’s College London, London, UK; 11 Department of Radiology, Beth Israel Deaconess Medical Center, Harvard Medical School, MA Boston, USA; 12 NIHR Clinical Research Facility, Lancashire Teaching Hospitals NHS Foundation Trust, Preston, UK; 13 Department of Cellular Pathology, Lancashire Teaching Hospitals NHS Foundation Trust, Preston, UK; 14 Department of Gynaeoncology, Imperial College Healthcare NHS Trust, London, UK; 15 Department of Cancer and Genomic Science, University of Birmingham and Pan Birmingham Gynaecological Oncology Centre, Midlands Metropolitan University Hospital, Birmingham, UK; 16 Wolfson Institute of Population Health, Queen Mary University of London, Charterhouse Square, London, UK; 17 Department of Gynaecological Oncology, Barts Health NHS Trust, London, UK; 18 UK Department of Health Services Research, London School of Hygiene & Tropical Medicine, London, UK; 19 Dept of Radiology, St Bartholomew’s Hospital, Barts Health NHS Trust, London, UK

**Keywords:** SUV_max_, ADC_mean_, PET/CT, [^18^F]FDG, [^18^F]fluoroethylcholine, Cervical cancer, Endometrial cancer, Lymph node metastasis

## Abstract

**Purpose:**

To evaluate the diagnostic performance of quantitative measures derived from [^18^F]FDG PET/CT, [^18^F]FEC PET/CT, and DW-MRI in the detection of lymph node metastases in endometrial and cervical cancer with comparison to standard visual PET analysis with histology as the reference standard.

**Methods:**

Subanalysis of quantitative data from the prospective multicentre MAPPING study. Nodal and tumour SUV_max_ from [^18^F]FDG PET/CT and [^18^F]FEC PET/CT and ADC_mean_ from DW-MRI were documented. Nodal-to-tumour ratios (NTR) and SUV_max_-to-ADC_mean_ ratio (STAR) were calculated. Optimal cut-offs of quantitative measures were compared to visual assessment on a regional basis using histopathology as the reference standard.

**Results:**

Scans from 112 patients (36 cervical and 76 endometrial cancers; 340 nodal regions) were eligible for quantitative image analysis. Lower ADC_mean_ on DW-MRI was observed in metastatic nodes for cervical cancer but not for endometrial cancer. Quantitative measures were significantly higher in malignant than benign nodal regions on [^18^F]FDG PET/CT and [^18^F]FEC PET/CT in endometrial cancer. SUV_max_ cut-offs showed similar performance to visual assessment in the diagnosis of metastatic lymph nodes in endometrial cancer whilst ADC_mean_ cut-offs showed significantly lower specificity than visual assessment. Interobserver agreement was excellent for SUV_max_ measurements on both [^18^F]FDG PET/CT and [^18^F]FEC PET/CT, but poor for ADC_mean_ on DW-MRI.

**Conclusion:**

Quantitative measures from [^18^F]FDG PET/CT, [^18^F]FEC PET/CT, or DW-MRI did not outperform visual assessment in the detection of nodal metastases in endometrial cancer. Therefore, the implementation of these quantitative measures as standalone diagnostic tools in routine clinical practice is not recommended.

**Supplementary Information:**

The online version contains supplementary material available at 10.1007/s00259-025-07587-3.

## Introduction

Nodal status for cervical and endometrial cancers is prognostic and highly important in determining therapeutic approach. Prognosis of node positive endometrial and cervical cancer is poor; the 5-year disease free survival in uterine-limited endometrial cancer is estimated at 90% for patients without lymph node metastasis, 60–70% for those with malignant pelvic lymph nodes and 30–40% with malignant para-aortic lymph nodes [1]. In cervical cancer, 5-year overall survival is estimated at 92% with localised disease and 58% with regional disease (including lymph nodes metastasis) [1]. Treatment options differ for patients with lymph node involvement. Patients with early localised cervical cancer typically receive surgery as their primary treatment whereas patients with lymph node involvement undergo primary chemoradiotherapy [2]. In endometrial cancer, most patients undergo hysterectomy and those at high risk of nodal involvement may undergo lymphadenectomy [3]. However, whilst lymphadenectomy is considered the most accurate technique for the detection of lymph node metastases, the therapeutic value is controversial as the procedure carries significant risks, cost, and has not been shown to reliably convey any significant survival benefit [4–6].

Magnetic resonance imaging (MRI) is considered the optimal imaging technique for local tumour staging of both cervical and endometrial cancers but detection of nodal involvement, using size criteria, is limited as tumour may have spread to non-enlarged nodes (micrometastases) and nodes can enlarge due to benign conditions [7, 8]. Beyond conventional anatomical imaging, both advanced MRI techniques, such as diffusion-weighted MRI (DW-MRI), and positron emission tomography (PET)-based molecular imaging offer complementary information about tissue microenvironment and metabolism that may improve the diagnosis of lymph node metastases.

Integrated PET/computed tomography (PET/CT) with [^18^F]fluoro-2-deoxy-D-glucose ([^18^F]FDG PET/CT), a glucose analogue, allows combined anatomical and molecular imaging of glucose metabolism within a tumour. [^18^F]FDG PET/CT has an established role in staging of locally advanced cervical cancers but its role in endometrial cancer is not so clear [2, 9]. Maximum standardised uptake value (SUV_max_) is a semi-quantitative measure derived from PET/CT imaging that represents the maximum level of tracer uptake within a region of interest (ROI) normalised for administered activity and body weight.

Radiolabelled choline ([^18^F]-fluoroethylcholine) PET/CT ([^18^F]FEC PET/CT) assesses cell membrane metabolism in vivo and has established use in detection of malignant lymph nodes in prostate cancer [10]. Despite promising early findings, prior to the MAPPING study no large prospective studies had reported the performance of [^18^F]FEC PET/CT in the diagnosis of nodal involvement in endometrial or cervical cancer.

DW-MRI detects changes in the diffusion properties of water in tissues. This is primarily influenced by cellular density and provides microstructure information regarding tumour tissues. Metastatic lymph nodes are expected to have higher cellular density than benign nodes. Apparent diffusion coefficient (ADC) from DW-MRI provides indirect quantitative assessment of cellular density.

The MAPPING study was a large, multicentre, prospective study with the primary aim of assessing the diagnostic accuracy of [^18^F]FDG PET/CT, [^18^F]FEC PET/CT and DW-MRI for the detection of nodal disease in surgically staged endometrial and cervical cancers [11]. Visual assessment of both PET/CT and DW-MRI scans was performed. This study showed that the sensitivity of [^18^F]FDG PET/CT, [^18^F]FEC PET/CT, and DW-MRI in the detection of lymph node metastases in endometrial and cervical cancer, was not sufficient to obviate the need for surgical nodal detection. However, the low false positive rate suggested that these scans could play a role in ruling out patients in challenging surgical decision-making cases and enable targeted, less extensive lymphadenectomy [11].

The utility of SUV_max_ from [^18^F]FDG PET/CT and ADC_mean_ from DW-MRI in the differentiation of benign and malignant nodes has been explored in retrospective or small prospective studies in endometrial and cervical cancer [12–22]. No studies have investigated the performance of SUV_max_ from [^18^F]FEC PET/CT in the detection of metastatic lymph nodes in endometrial or cervical cancer. Furthermore, a comprehensive comparison of the diagnostic performance of these measures with visual assessment has not yet been undertaken.

The present study aims to investigate the diagnostic utility of quantitative measurements from [^18^F]FDG PET/CT, [^18^F]FEC PET/CT, and DW-MRI in the differentiation of metastatic and benign lymph nodes in surgically staged endometrial and cervical cancer, using histopathology as the reference standard.

## Methods

### Patients

This study forms a retrospective secondary analysis of data from the MAPPING study: a multicentre, prospective study evaluating the diagnostic accuracy of [^18^F]FDG PET/CT, [^18^F]FEC PET/CT, and DW-MRI in the detection of nodal metastases. A full summary of the Materials and Methods can be found in Rockall et al. [11].

Ethics approval, Administration of Radioactive Substances Advisory Committee (ARSAC) licence, and Medicines and Healthcare products Regulatory Agency (MHRA) approvals were obtained (Research Ethics Committee reference number 11/LO/1465). Participants were prospectively recruited from 5 gynae-oncology tertiary UK referral centres between October 2012 and July 2017. Eligible patients were over the age of 18 years with newly diagnosed, histologically confirmed cervical or endometrial cancer, and were eligible and fit for surgical lymphadenectomy. All participants gave written informed consent. Age, tumour grade, pre- and post-treatment Federation of Gynaecology and Obstetrics (FIGO) stage (2009; from MRI) and the final treatment received were collected.

### [^18^F]FDG PET/CT and [^18^F]FEC PET/CT protocol

[^18^F]FDG and [^18^F]FEC PET/CT scans were acquired using a standardised protocol based on UK National Cancer Research Institute (NCRI) PET Research Network guidance at accredited centres as previously described [11]. In brief, a standardised PET/CT protocol was established to minimise differences between scanners and phantom PET harmonisation was undertaken, with data sent to UK PET Core Lab to ensure protocol adherence. Participants fasted for ≥ 4 h prior to [^18^F]FDG injection (median 370 MBq) and scanning was only performed if blood glucose was < 10 mmol/L. Fasting was not required prior to [^18^F]FEC injection (median 293 MBq). Patients were asked to void their bladder before both scans. Diuretics were not administered. Low dose non-contrast CT acquisitions were acquired. All scans were acquired from base of skull to upper thighs as 3D acquisitions with TOF if available.

### DW-MRI protocol

Standard of care conventional MRI scan for staging was performed as per local protocol which included a minimal dataset of MR sequences. The MRI field of view included the pelvis and para-aortic regions up to the level of the left renal vein. For nodal evaluation, readers had a minimal dataset that included axial T1, axial T2 and axial diffusion weighted MRI (b values 0, 300, 600, 900 and 1200) with associated calculated ADC maps, following optimisation with ice-water phantom.

### Surgical staging and pathologic evaluation

The primary reference standard was confirmed nodal histology by lymphadenectomy as per the MAPPING study protocol. Anatomical location, presence of metastatic disease, and lymph node dimensions in each region (right pelvis, left pelvis, and para-aortic) were recorded. A region was determined to be positive for nodal metastases with the presence of at least one malignant node from that region at histology. Prior to lymphadenectomy, the surgeon received information about the location of any suspicious lymph nodes to maximize the probability of identifying all nodes suspected to be positive on imaging studies. The surgeon labelled the sites of resected nodes according to a predefined protocol. All retrieved nodes were analysed by an expert gynae-oncology histopathologist who was blinded to the imaging findings but aware of the histopathology of the primary tumour pre-operative biopsy. When imaging indicated nodal involvement, but histopathological examination yielded negative results, additional reviews were conducted to prevent an unreliable reference standard due to the potential that the node had not been successfully removed during surgery: Initially, expert consensus review of any postoperative imaging was performed to confirm complete retrieval of all suspicious nodes during the surgical procedure. If the suspicious node remained in situ, the case was excluded from analysis using the primary reference standard since histological correlation remained uncertain. Additionally, the most suspicious node underwent ultra-sectioning and immunostaining to ensure very high sensitivity for detecting micrometastases.

### Image evaluation

All images were read by two expert central MRI and PET readers; the central reads were co-ordinated through the UK PET Core Lab and the trials unit. Readers scored nodes by visual assessment on a 6-point confidence score (1 – definitely benign, 6 – definitely malignant); nodes with a score of 5 or 6 were classed as malignant. A third central PET reader reviewed all PET measurements and recorded additional readings of any suitable nodes, as the original case report form (CRF) required readers to only document the SUV_max_ of suspicious nodes. All readers were accredited radiologists that were core members of the gynae-oncology multi-disciplinary team and/or PET/CT experts. Readers were aware of the clinical diagnosis (endometrial or cervical cancer) from the pre-operative biopsy results, as per standard clinical practice. Readers were blinded to other imaging studies and the final nodal histology.

### [^18^F]FDG PET/CT and [^18^F]FEC PET/CT image evaluation

The anatomical location of any focally increased tracer uptake that was higher than background adjacent tissue corresponding to a node of any size on the CT was recorded. An elliptical ROI was manually placed on the slice with the highest activity of the most avid node for each region (right/left pelvis and para-aortic) and nodal SUV_max_ was recorded. In regions with no nodes demonstrating tracer uptake above background on PET, if a node greater than 8 mm long-axis was visible on CT but not demonstrating tracer uptake above background, the SUV_max_ of the node was recorded using the CT for guidance to include more potential benign nodes for analysis and reduce selection bias. SUV_max_ of the primary tumour was also measured by a single central expert PET reader by placing a ROI over the primary tumour on the slice with the highest activity on a dedicated PET/CT workstation (Hermes Medical Solutions).

### DW-MRI image evaluation

Readers reviewed the DW-MRI in conjunction with standard MRI sequences. Nodes were identified on the high b-value (b = 1200 mm^2^/s) DW-MRI as non-continuous high signal intensity (SI) round or ovoid structures that corresponded with a node on the anatomical images. Short-axis nodal diameter was measured and, for nodes greater than 5 mm short-axis diameter, ADC_mean_ of the most restricted node in each nodal region was recorded. ADC_mean_ was measured by manually placing a regular ROI, avoiding the margin, and noted with anatomical location. The ADC_mean_ of the primary tumour, if visible, was also measured by both readers in an identical fashion. Units for ADC_mean_ measurements are 10^−6^ mm^2^/s.

### Dataset

In cases where both readers provided measurements in a region, the readings were averaged. Where only one reader provided a measurement in a region, this measurement was used. Where no central readers provided a measurement in a region, the measurement of the third external reader was used. The mean SUV_max_ from [^18^F]FDG PET/CT and [^18^F]FEC PET/CT, and ADC_mean_ of the primary tumour from both readers was calculated and included in the final analysis. Reads in regions that had corresponding nodal histology were included in the analysis.

Where data for both primary tumour and nodal regions were recorded, nodal-to-tumour ratio (NTR) for [^18^F]FDG PET/CT and [^18^F]FEC PET/CT was calculated by dividing the SUV_max_ of the nodal region by the SUV_max_ of the primary tumour. For DW-MRI, NTR was calculating by dividing nodal ADC_mean_ by the ADC_mean_ of the primary tumour. SUV_max_-to-ADC_mean_ ratio (STAR) was calculated by dividing nodal SUV_max_ by nodal ADC_mean_ for [^18^F]FDG PET/CT and [^18^F]FEC PET/CT. Direct measurements from scans are termed ‘raw’ measurements, whilst NTR and STAR are termed ‘calculated’ measurements.

### Statistical analysis

All analyses were undertaken in R (version 4.4.1) with packages from Bioconductor (version 3.20).

Patients were stratified into two cohorts by their diagnosis of endometrial or cervical cancer. The Mann-Whitney U-test was selected to compare quantitative measures in histologically confirmed benign versus malignant lymph nodes. One-way ANOVA with Tukey’s post-hoc test was used to determine significance when comparing left pelvic, right pelvic, and para-aortic regions. P-values were adjusted using the false-discovery rate (FDR) method.

Pairwise Pearson correlation coefficients were calculated between raw quantitative measures and p-values were adjusted using the FDR method. Additionally, Pearson correlation coefficients were calculated between patient age, body mass index (BMI), and FIGO stage and quantitative measures and p-values were adjusted using the FDR method.

Receiver operating characteristic (ROC) analysis was applied, using the ‘pROC’ package, for each quantitative measure to generate ROC curves [23].

Cut-off estimation was performed using the ‘cutpointr’ package [24]. Direction was set to greater than or equal to for all measures except ADC_mean_ and ADC_mean_ NTR where direction was set to less than or equal to. Cut-offs were selected through maximisation of the F_β_ score. The F_β_ score is the harmonic mean of sensitivity and positive predictive value (PPV). By controlling the hyperparameter β, the relative importance of sensitivity and PPV can be controlled: $\:\beta\:=1$ balances sensitivity with PPV, $\:\beta\:>1$ favours sensitivity over PPV, and $\:\beta\:<1$ favours PPV over sensitivity.

$$\:{F}_{\beta\:}=\left(1+{\beta\:}^{2}\right)\cdot\:\frac{\mathrm{PPV}\cdot\:\mathrm{sensitivity}}{\left({\beta\:}^{2}\cdot\:\mathrm{PPV}\right)+\mathrm{sensitivity}}$$

To fairly compare quantitative measurements with visual assessments (which were performed blind to nodal histology), leave-one-out cross-validation was used to evaluate each quantitative measure’s diagnostic performance on unseen data. For each sample, optimal cut-offs were determined using all other samples, with the held-out sample then classified based on this threshold. Visual assessments were paired with quantitative predictions on a regional basis. Diagnostic performance metrics – including sensitivity, specificity, positive predictive value (PPV), negative predictive value (NPV), and F1-score – were calculated for both visual assessment and quantitative predictions. Confidence intervals (95%) were established through 1000 bootstrap iterations using the percentile method. The median optimal cut-off value across all cross-validation iterations is reported.

Interobserver agreement between all three readers for [^18^F]FDG PET/CT and [^18^F]FEC PET/CT and the two central readers for DW-MRI was evaluated by calculating intraclass correlation coefficients (ICC) for endometrial and cervical cancer cohorts separately using ‘irr’ package. ICC for the primary tumour ADC_mean_ measurements were calculated. A one-way model was used and consistency between readers was evaluated. Confidence level of the interval was set at 95%.

## Results

### Demographics

**Fig. 1:**
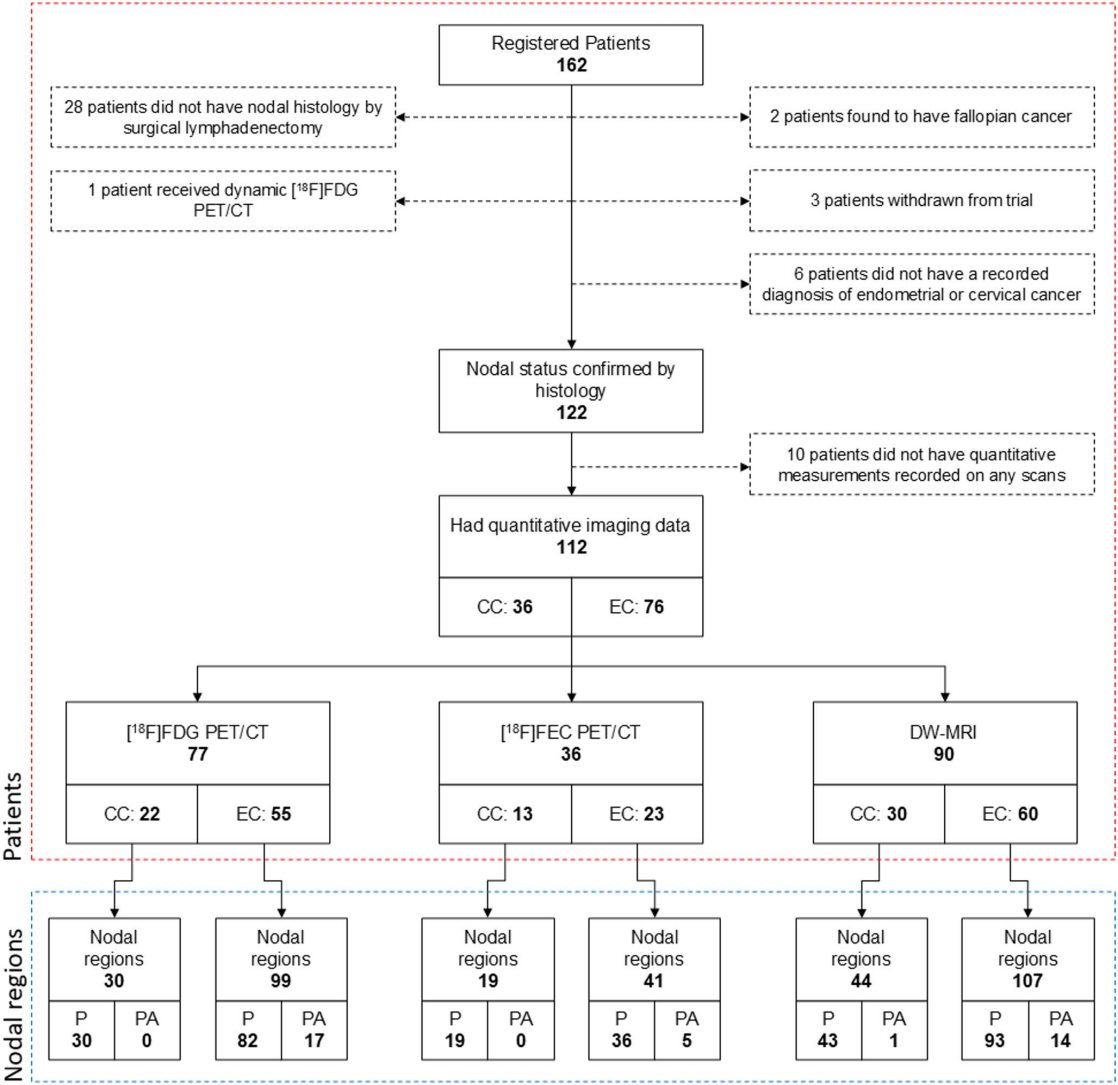
Study consort diagram showing patient enrolment and eligibility for analysis. EC, endometrial cancer; CC, cervical cancer; P, pelvic nodal regions; PA, para-aortic nodal regions. Red region represents number of patients, blue region represents number of regions

Among 162 patients recruited to the MAPPING study, a total of 112 patients were eligible for analysis: 36 cervical cancer patients and 76 endometrial cancer patients. Number of nodal regions available for analysis are summarised in Fig. 1. 86% of cervical cancer patients were diagnosed as FIGO stage 1B1 and no patients in this cohort were diagnosed with FIGO stage greater than 2B. In the endometrial cancer cohort, FIGO stage ranged from 1 A to 4B. The demographics of patients eligible for analysis are summarised in Supplementary Table 1.

### Quantitative measures in benign and malignant lymph nodes

**Fig. 2:**
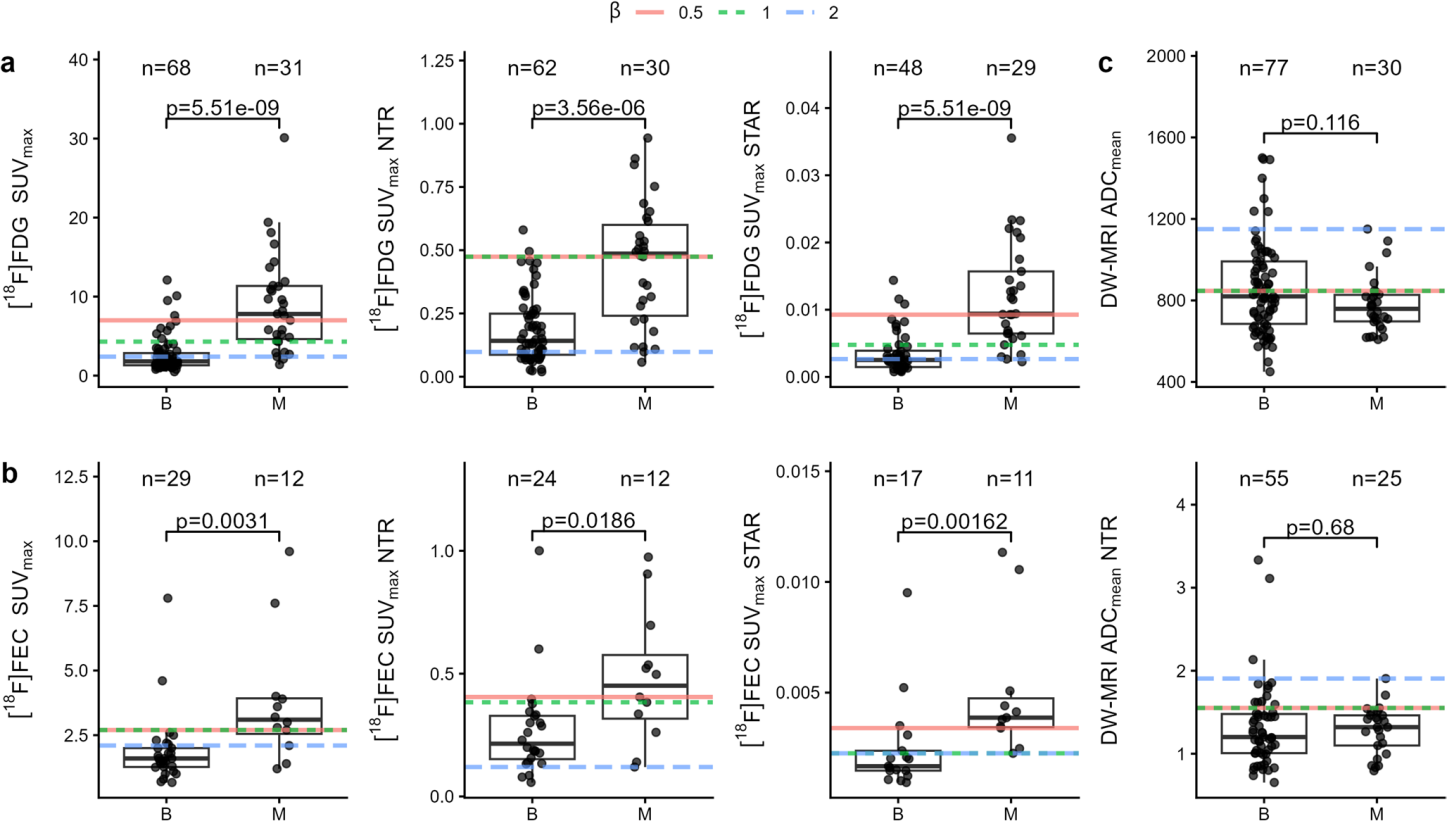
Raw and calculated quantitative measures from benign (B) and malignant (M) nodal regions in endometrial cancer for (**a**) [^18^F]FDG PET/CT and (**b**) [^18^F]FEC PET/CT and (**c**) DW-MRI. Numbers of nodal regions and adjusted *p*-values are represented above each boxplot. Coloured lines represent optimal cut-offs at different values of β

No significant difference was observed in raw quantitative measures between left, right, and para-aortic regions. On [^18^F]FDG PET/CT, nodal SUV_max_ was significantly higher in endometrial compared to cervical cancers (*p* = 0.016); the analysis was therefore stratified by cancer type with pelvic and para-aortic regions grouped (Supplementary Fig. 1). Nodal SUV_max_, NTR, and STAR were all significantly higher in malignant than benign nodal regions on [^18^F]FDG PET/CT and [^18^F]FEC PET/CT in endometrial cancer (Fig. 2). ADC_mean_ and ADC_mean_ NTR was not significantly different between benign and malignant nodal regions in endometrial cancer.

**Fig. 3:**
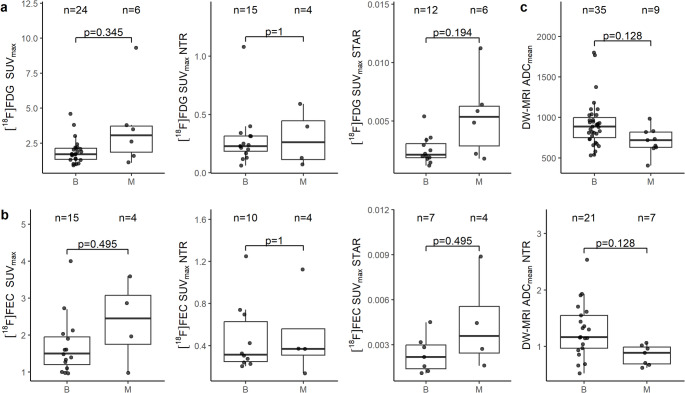
Raw and calculated quantitative measures from benign (B) and malignant (M) nodal regions in cervical cancer for (**a**) [^18^F]FDG PET/CT and (**b**) [^18^F]FEC PET/CT and (**c**) DW-MRI. Numbers of nodal regions and adjusted *p*-values are represented above each boxplot

No calculated or raw measures were significantly different between benign and malignant nodal regions on [^18^F]FDG PET/CT and [^18^F]FEC PET/CT in cervical cancer, although median raw measures were consistently higher in malignant regions (Fig. 3). ADC_mean_ and ADC_mean_ NTR were significantly lower in malignant regions in cervical cancer.

SUV_max_ of the primary tumour was significantly elevated in endometrial cancer patients with metastatic lymph nodes compared to endometrial cancer patients with benign nodes on [^18^F]FDG PET/CT (*p* < 0.01) but not on [^18^F]FEC PET/CT (Supplementary Fig. 2). Additionally, no significant difference was observed in ADC_mean_ of the primary tumour between these patients. No significant difference in any quantitative measure was found in primary tumours of the cervix.

Pearson correlation coefficients were calculated between quantitative measures in endometrial cancer (Supplementary Fig. 3). SUV_max_ of nodal regions were highly correlated between [^18^F]FDG PET/CT and [^18^F]FEC PET/CT. [^18^F]FDG PET/CT SUV_max_ was weakly negatively correlated with ADC_mean_. Measures from primary tumours were correlated.

The higher SUV_max_ on [^18^F]FDG PET/CT and [^18^F]FEC PET/CT and lower ADC_mean_ between benign and malignant lymph nodes were generally consistent across patient histology and FIGO stage (Supplementary Figs. 4 & 5).

### Diagnostic performance of quantitative measures

**Fig. 4:**
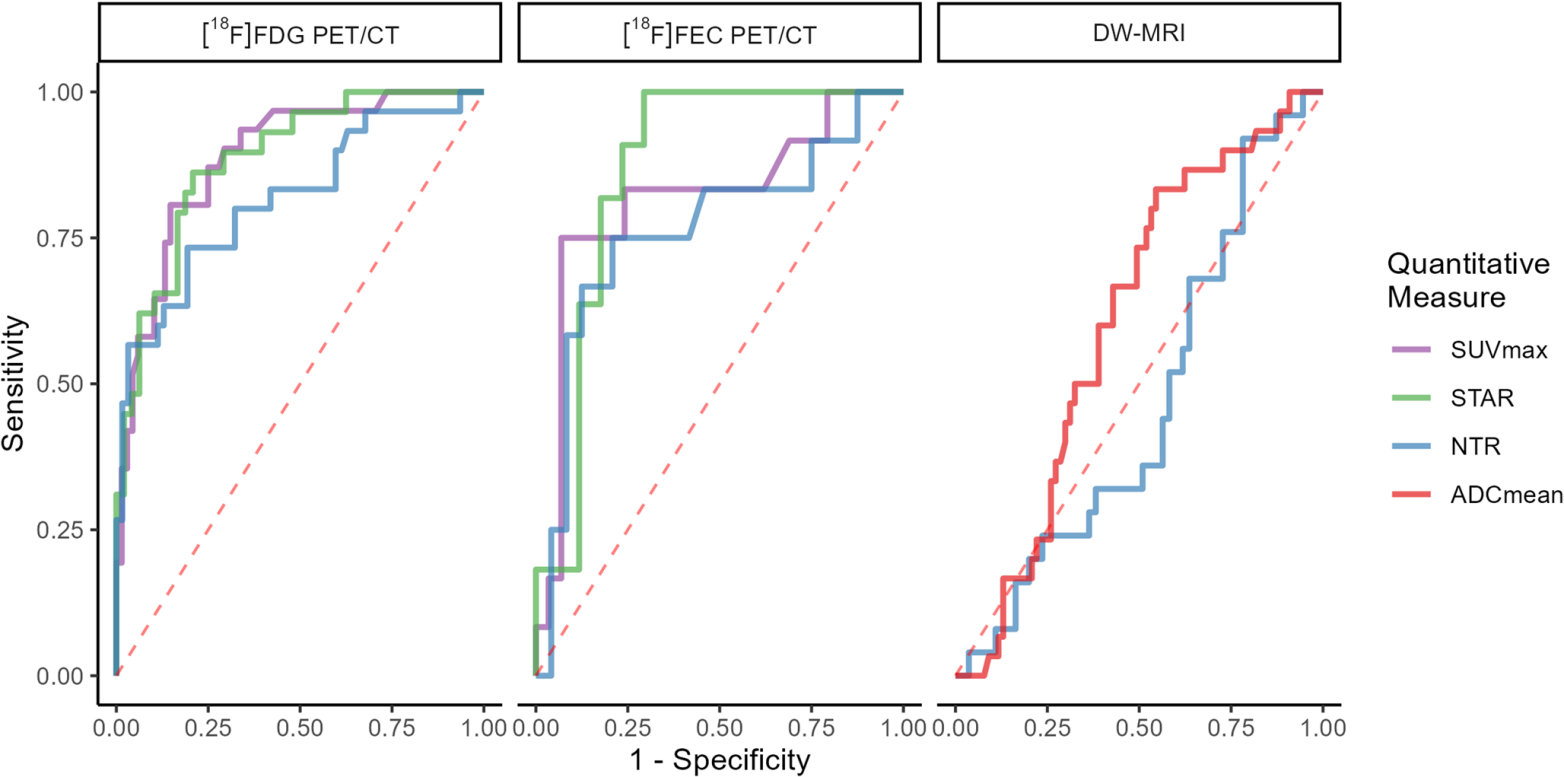
ROC curves for quantitative measures from [^18^F]FDG PET/CT, [^18^F]FEC PET/CT, DW-MRI in endometrial cancer. Line colour represents a quantitative measure for that imaging modality

In endometrial cancer, ROC analysis showed SUV_max_ and STAR performed equivalently on [^18^F]FDG PET/CT (Fig. 4). Whilst lower sample numbers limited analysis, [^18^F]FEC PET/CT showed similar performance to [^18^F]FDG PET/CT. DW-MRI performed poorly across all cut-offs.

**Table 1:** Diagnostic performance of all quantitative measures and visual assessment in endometrial cancer

				Quantitative								
		OC	NR	TP	FN	TN	FP	Sensitivity (%)	Specificity (%)	PPV (%)	NPV (%)	F1 (%)
[^18^F]FDG PET/CT	SUV_max_	4.3	99	24	7	58	10	77.4 (61.8–91.3)	85.3 (76.5–93.0)	70.6 (54.2–84.6)	89.2 (80.6–96.4)	73.8 (60.5–83.9)
	STAR	0.00477	77	23	6	38	10	79.3 (63.3–93.3)	79.2 (67.4–89.7)	69.7 (52.9–84.8)	86.4 (75.0–95.7.0.7)	74.2 (59.7–85.7)
	NTR	0.474	92	16	14	50	12	53.3 (35.3–70.3)	80.6 (71.2–90.5)	57.1 (40.0–75.8.0.8)	78.1 (68.2–87.7)	55.2 (40.0–69.7.0.7)
[^18^F]FEC PET/CT	SUV_max_	2.7	41	8	4	27	2	66.7 (36.4–91.7)	93.1 (82.4–100.0)	80.0 (50.0–100.0.0.0)	87.1 (72.7–97.0)	72.7 (44.4–91.7)
	STAR	0.00226	28	9	2	12	5	81.8 (57.1–100.0)	70.6 (47.4–92.3)	64.3 (38.5–88.9)	85.7 (64.3–100.0)	72.0 (48.0–88.9.0.9)
	NTR	0.384	36	7	5	19	5	58.3 (28.6–85.7)	79.2 (60.9–95.2)	58.3 (28.6–87.5)	79.2 (60.9–94.7)	58.3 (30.0–80.0)
DW-MRI	ADC_mean_	847	107	24	6	35	42	80.0 (64.7–93.3)	45.5 (33.3–57.1) *	36.4 (24.3–48.3)	85.4 (73.5–95.1)	50.0 (36.4–62.1)
	NTR	1.55	80	22	3	12	43	88.0 (72.7–100.0)	21.8 (11.3–33.3) *	33.8 (22.8–45.6)	80.0 (56.5–100.0)	48.9 (35.3–60.7)
				**Visual**								
			NR	TP	FN	TN	FP	Sensitivity (%)	Specificity (%)	PPV (%)	NPV (%)	F1 (%)
[^18^F]FDG PET/CT			99	26	5	60	8	83.9 (70.4–96.4)	88.2 (80.9–95.5)	76.5 (62.1–89.7)	92.3 (84.8–98.4)	80.0 (69.0–90.0)
			77	25	4	41	7	86.2 (72.7–96.8)	85.4 (75.0–95.5.0.5)	78.1 (63.3–92.3)	91.1 (82.6–98.0)	82.0 (70.4–91.8)
			92	25	5	55	7	83.3 (68.0–96.2.0.2)	88.7 (80.0–96.4.0.4)	78.1 (63.0–91.9.0.9)	91.7 (83.9–98.3)	80.6 (68.8–90.0)
[^18^F]FEC PET/CT			41	8	4	26	3	66.7 (36.4–91.7)	89.7 (76.7–100.0)	72.7 (42.9–100.0)	86.7 (72.4–96.9)	69.6 (40.0–88.2.0.2)
			28	8	3	14	3	72.7 (44.4–100.0)	82.4 (64.3–100.0)	72.7 (44.4–100.0)	82.4 (60.0–100.0.0.0)	72.7 (47.1–90.9)
			36	8	4	21	3	66.7 (36.4–92.3)	87.5 (72.0–100.0.0.0)	72.7 (42.9–100.0)	84.0 (68.2–96.3)	69.6 (42.9–88.9)
DW-MRI			107	23	7	71	6	76.7 (61.1–91.4)	92.2 (85.5–97.5) *	79.3 (64.1–93.7)	91.0 (84.0–97.3.0.3)	78.0 (65.1–89.2)
			80	20	5	51	4	80.0 (62.5–95.6)	92.7 (85.7–98.2) *	83.3 (66.7–95.8)	91.1 (82.5–98.1)	81.6 (68.2–91.5)

Visual assessments of nodal regions were matched to quantitative measurements and diagnostic performances estimated with β = 1. Diagnostic performance is shown with 95% confidence intervals in brackets. Asterisks represent FDR-adjusted *p*-values on McNemar’s test of sensitivity and specificity; *, *p*<0.001. *OC* optimal cut-off, *NR* number of regions, *TP* true positive, *FN* false negative, *TN* true negative, *FP* false positive

**Fig. 5:**
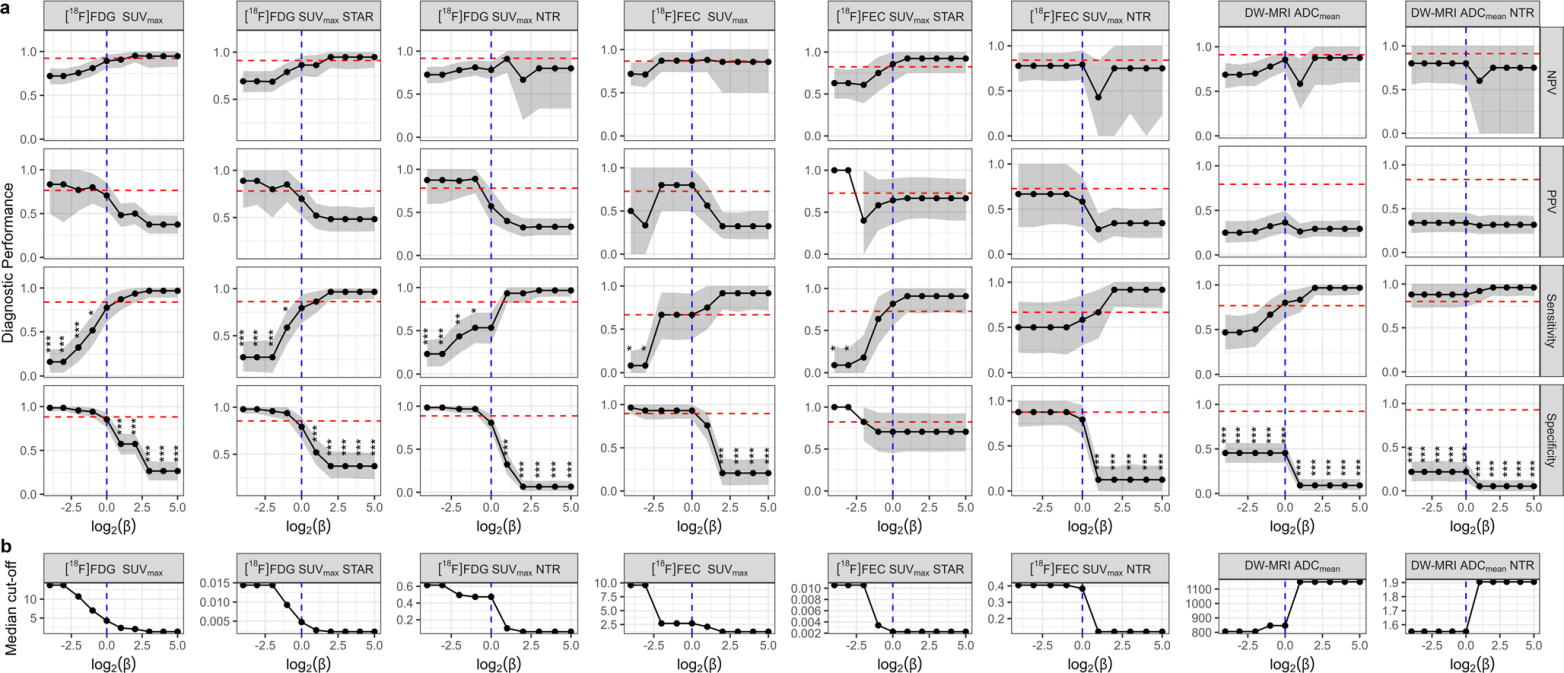
Estimates of diagnostic performance across different β-values and corresponding optimal cut-offs for each imaging modality in endometrial cancer. **a** Diagnostic performance across a range of β-values. The red line denotes performance of visual assessment of matched regions for each modality; the blue line denotes β = 1. Asterisks represent FDR-adjusted p-values from McNemar’s test of sensitivity and specificity compared to visual assessment. *, *p* < 0.05; **, *p* < 0.01; ***, *p* < 0.001. **b** Median optimal cut-off determined for a range of β-values

On DW-MRI, the optimal ADC_mean_ for detecting malignant nodes cut-off was found to be 847 × 10^−6^ mm^2^/s (Table 1). This cut-off showed moderate sensitivity, albeit with a low positive predictive value, and a low specificity (80.0%, 36.4%, 45.5% respectively). The specificity of both the ADC_mean_ and ADC_mean_ NTR cut-off was significantly lower than visual assessment (*p* < 0.001). The optimal SUV_max_ cut-off identified from [^18^F]FDG PET/CT in the endometrial cancer cohort was 4.3 and is associated with moderate specificity and sensitivity (77.4% and 85.3%, respectively). Finally, an optimal cut-off of 2.7 SUV_max_ on [^18^F]FEC PET/CT was associated with low sensitivity, high specificity, and moderate positive and negative predictive values (66.7%, 93.1%, 80.0%, 85.7% respectively).

No raw measures showed significantly higher diagnostic performance compared to visual assessment.

Calculated measures, STAR and NTR, showed similar or slightly decreased diagnostic performance, and all had overlapping 95% confidence intervals, with respect to the raw measurements.

As expected, increasing β-values decreased SUV_max_ cut-offs identified on [^18^F]FDG PET/CT and [^18^F]FEC PET/CT and increased ADC_mean_ cut-offs, favouring sensitivity and penalising PPV and specificity (Fig. 5). Visual assessment had significantly higher specificity across all values of β for ADC_mean_ and ADC_mean_ NTR. Whilst sensitivity improved for all quantitative measures with increasing β, no level of β showed a significant improvement in sensitivity compared to visual assessment. On the other hand, low values of β produced SUV_max_ cut-offs with significantly worse sensitivity compared to visual assessment and high values of β produced significantly worse specificity for all measures except STAR on [^18^F]FEC PET/CT. At high β-values, low STAR cut-offs on both [^18^F]FDG PET/CT and [^18^F]FEC PET/CT showed high sensitivity (96.6% (95% CI [88.0–100.0.0.0]) and 90.9% (95% CI [0.70–100.0]), respectively) and low-to-moderate PPV (48.3% (95% CI [35.6–61.9]) and 66.7% (95% CI [41.7–92.3]), respectively).

In cervical cancer, wide confidence intervals were observed for all quantitative measures and visual assessment due to the low number of regions available for analysis and low prevalence of metastatic nodes (Supplementary Table 2).

### Interobserver agreement

Intraclass correlation coefficients (ICC) were calculated for each imaging modality for endometrial and cervical cancer separately (Table 2). SUV_max_ measurement on both [^18^F]FDG PET/CT and [^18^F]FEC PET/CT in endometrial cancer had excellent interobserver agreement at 0.976 and 0.954, respectively. However, ADC_mean_ measurement on DW-MRI had poor interobserver agreement at 0.580. In cervical cancer, interobserver agreement was lower for all quantitative measurements, especially for lymph node and primary tumour ADC_mean_ measurements on DW-MRI.

**Table 2:** Intraclass correlation coefficients (ICC) between reader measurements of quantitative measures in lymph nodes in endometrial and cervical cancer. PT, primary tumour

Cancer	Method	Quantitative Measure	ICC	95% CI
Endometrial	[^18^F]FDG PET/CT	SUV_max_	0.976	0.964–0.984
	[^18^F]FEC PET/CT	SUV_max_	0.954	0.911–0.979
	DW-MRI	ADC_mean_	0.580	0.385–0.725
	DW-MRI (PT)	ADC_mean_	0.781	0.501–0.914
Cervical	[^18^F]FDG PET/CT	SUV_max_	0.741	0.442–0.919
	[^18^F]FEC PET/CT	SUV_max_	0.891	0.697–0.974
	DW-MRI	ADC_mean_	0.477	0.023–0.771
	DW-MRI (PT)	ADC_mean_	−0.016	−0.648-0.652

## Discussion

This study represents a retrospective secondary analysis of the largest prospective multicentre study assessing the use of quantitative measures from [^18^F]FDG PET/CT, [^18^F]FEC PET/CT, and DW-MRI in the diagnosis of lymph node metastases in endometrial and cervical cancer with nodal histology as the reference standard to-date. Our aim was to assess if quantitative parameters, alone or in combination, could improve the diagnostic performance for nodal involvement compared to standard visual assessment.

SUV_max_ was significantly elevated in metastatic lymph nodes compared to benign on both [^18^F]FDG PET/CT and [^18^F]FEC PET/CT. Lower ADC_mean_ on DW-MRI was observed in metastatic nodes for cervical cancer but not for endometrial cancer. SUV_max_ cut-offs on [^18^F]FDG PET/CT and [^18^F]FEC PET/CT showed similar performance to visual assessment in the diagnosis of metastatic lymph nodes in endometrial cancer, with no significant difference in sensitivity or specificity. ADC_mean_ cut-offs on DW-MRI showed significantly lower specificity than visual assessment. Interobserver agreement was excellent for SUV_max_ measurements on both [^18^F]FDG PET/CT and [^18^F]FEC PET/CT, but poor for ADC_mean_ on DW-MRI.

A range of *β-*values for each quantitative measure were assessed to demonstrate how the optimal cut-off varies depending on the priority of sensitivity and PPV. In this context, *β-*values > 1 (which favour sensitivity over PPV) may be considered more appropriate, as the consequences of missing a true positive are more serious than those of a false positive, which can often be resolved through further testing. Crucially, however, no *β*-value was sufficient to improve the performance of a quantitative measure to represent a significant improvement over visual assessment.

Studies investigating nodal ADC_mean_ in endometrial and cervical cancer have reported heterogeneous results; some studies found ADC_mean_ was lower in malignant nodes and others reported no significant difference between malignant and benign nodes [12, 25, 26]. ADC_mean_ cut-offs on DW-MRI have been shown to be similar for both endometrial and cervical cancer in previous studies (780–1,150 × 10^−6^ mm^2^/s), displaying moderate sensitivity and specificity, and are in line with the cut-off of 847 × 10^−6^ mm^2^/s identified presently in the endometrial cancer cohort [12–19]. Whilst elevated ADC_mean_ cut-offs were highly sensitive, they lacked specificity and PPV across the range of β-values. No significant difference in ADC_mean_ between benign and malignant nodes and associated poor diagnostic performance of this measurement, particularly the low specificity, suggests it lacks utility in differentiating benign from malignant nodes in endometrial cancer. The lower specificity of ADC_mean_ cut-offs compared to visual assessment is likely due to visual assessment simultaneously considering morphological features of the nodes; DW-MRI helps to localise both benign and malignant nodes and is coupled with assessment of morphological features (nodal size, signal intensity, and necrosis) on other sequences.

Despite a significant difference in ADC_mean_ between benign and malignant nodes in cervical cancer, estimations of diagnostic performance were not possible in the cervical cancer cohort due to low numbers of patients and low prevalence of nodal disease. This is evidenced by the wide confidence intervals for quantitative measures and visual assessment, showing the data is insufficient to reliably estimate the diagnostic performance of these in this cohort. The low prevalence of metastatic lymph nodes reflects standard clinical practice whereby patients with clear nodal involvement undergo chemoradiation therapy rather than lymphadenectomy, limiting the numbers of patients with histological reference standard.

In general, [^18^F]FDG PET/CT for nodal staging by visual assessment in cervical and endometrial cancers has high specificity (93%−97%) but lower sensitivity (63%−73%), particularly in early stage disease [27, 28]. Whilst several studies have found elevated SUV_max_ in metastatic lymph nodes from gynaecological cancers on [^18^F]FDG PET/CT, the potential of SUV_max_ cut-offs has not been fully explored [20, 22]. Kim et al. reported an optimal SUV_max_ cut-off of 2.8 in endometrial cancer with a respective sensitivity and specificity of 69% and 92% whilst the SENTIREC-Endo study reported an SUV_max_ cut-off of 3.95 with a respective sensitivity and specificity of 47% and 97% [20, 29]. Despite SUV_max_ being significantly elevated on [^18^F]FDG PET/CT in metastatic lymph nodes, many nodal regions exhibited SUV_max_ levels above the 4.3 SUV_max_ cut-off with benign histology suggesting benign processes can also elevate SUV_max_ in lymph nodes. Various confounding factors can influence SUV_max_ measurement including patient factors (serum glucose concentrations, patient body habitus, or presence of any local inflammatory processes) and technical factors (different scanners and reconstructions), although this was limited in this study by standardising protocols and quality assurance by the core PET laboratory.

No studies have investigated the performance of SUV_max_ from [^18^F]FEC PET/CT in the detection of metastatic lymph nodes in endometrial or cervical cancer. A recent prospective study on breast cancer patients by Clauser et al. showed that nodal SUV_max_ was significantly higher in malignant than benign lymph nodes [30]. Whilst low sample sizes limit the confidence of findings in [^18^F]FEC PET/CT, the strong correlation between [^18^F]FDG PET/CT and [^18^F]FEC PET/CT measurements suggests the measurements derived from these modalities are intrinsically related and similar diagnostic performance may be expected. The 2.7 SUV_max_ cut-off showed high specificity and NPV, suggesting it could be useful in ruling patients out of lymphadenectomy in challenging surgical planning decisions although the high cost of false negatives should be considered. Given the previously mentioned technical factors confounding SUV_max_ measurement, further studies with larger sample sizes would be required to reliably estimate the diagnostic performance of SUV_max_ cut-offs from [^18^F]FEC PET/CT across different scanning protocols.

Inverse correlations between SUV_max_ and ADC_mean_ and ADC_min_ have been documented [31, 32]. Primary tumour STAR has been reported to predict pelvic nodal involvement in a small retrospective staging [^18^F]FDG PET/MRI cervical cancer study [33]. NTR has been reported to improve the pre-operative staging accuracy of [^18^F]FDG PET/CT in detecting mediastinal nodal involvement in non-small cell lung cancer [34]. However, NTR and STAR have not been explored in gynaecological cancers to date. In the present analysis, NTR and STAR did not show significant improvements in diagnostic performance over visual assessment in any of the three imaging modalities. However, at high β-values, low STAR cut-offs on both [^18^F]FDG PET/CT and [^18^F]FEC PET/CT showed high sensitivity and moderate PPV and could therefore be a useful measure to ensure nodal metastasis without excessive false positives.

Excellent intraclass correlation coefficients (ICC) between readers on [^18^F]FDG PET/CT and [^18^F]FEC PET/CT suggests the method of measuring SUV_max_ is more robust than measuring ADC_mean_ on DW-MRI. As ADC_mean_ averages voxels across a reader-defined region-of-interest, it is much more susceptible to differences in reader bias compared to SUV_max_ and is likely the reason for lower interobserver agreement observed for DW-MRI. Future studies may be interested in alternative measures of ADC, such as ADC_min_. Interobserver agreement has been shown to be high for visual assessment, with Cohen’s kappa statistics, reported by Rockall et al., of 0.86, 0.79, and 0.70 for [^18^F]FDG PET/CT, [^18^F]FEC PET/CT, and DW-MRI, respectively [11].

As highlighted in the original MAPPING study, the size of the [^18^F]FEC PET/CT patient cohorts were limited by logistical challenges in tracer production. Regarding the present analysis, as readers were blinded between PET/CT and MRI it was not feasible to ensure measurements were taken from the same node. Additionally, as lymph nodes are generally removed en bloc and lymph nodes are histologically characterised post-surgery to obtain a surgical reference standard, it is difficult to ensure that nodes measured on imaging align with nodes identified by histology. Therefore, there is a possibility readers and surgeons identified different lymph nodes in the same region. Importantly, to ensure the highest likelihood of correlating suspected nodes based on imaging tests, surgeons were made aware of the position of suspicious lymph nodes prior to lymphadenectomy, and a rigorous procedure was implemented to robustly align uncertain imaging findings and histopathological characterisation.

## Conclusion

Despite statistically significant differences in SUV_max_ between malignant and benign lymph nodes on both [¹⁸F]FDG PET/CT and [¹⁸F]FEC PET/CT, the optimized cut-offs for quantitative measures across [¹⁸F]FDG PET/CT, [¹⁸F]FEC PET/CT, and DW-MRI failed to demonstrate meaningful improvements in diagnostic performance compared to expert visual assessment for detecting metastatic lymph nodes in endometrial cancer. Consequently, these findings do not support implementing these quantitative measures as standalone diagnostic tools in routine clinical practice.

## Supplementary Information

Below is the link to the electronic supplementary material.

Supplementary Material 1 (DOCX 549 KB)

## Data Availability

Code and the datasets analysed during the current study are available upon reasonable request to the principal investigator of the MAPPING study.
