# Supplementary material for: Diagnostic performance of quantitative measures from [18F]FDG PET/CT, [18F]FEC PET/CT, and DW-MRI in the detection of lymph node metastases in endometrial and cervical cancer: data from the MAPPING study
Source: Eur J Nucl Med Mol Imaging. 2025 Nov 3;53(4):2414–26. doi: 10.1007/s00259-025-07587-3 (PMC12920754; doi:10.1007/s00259-025-07587-3)
Supplement: Supplementary file 1 — Supplementary Material 1 (DOCX 549 KB) [file 259_2025_7587_MOESM1_ESM.docx]

Diagnostic performance of quantitative measures from [^18^F]FDG PET/CT, [^18^F]FEC PET/CT, and DW-MRI in the detection of lymph node metastases in endometrial and cervical cancer: data from the MAPPING study

Ben G. King^1^, Nishat Bharwani^2,3^, William Wilson^4^, Gary J.R. Cook^5^, Aslam Sohaib^6^, Marielle Nobbenhuis^7^, Victoria Warbey^8^, Marc E. Miquel^9,10^, Dow-Mu Koh^6^, Katja N De Paepe^11^, Pierre Martin-Hirsch^12^, Sadaf Ghaem-Maghami^3^, Christina Fotopoulou^3,14^, Helen Stringfellow^13^, Sudha Sundar^15^, Ranjit Manchanda^16,17,18^, Anju Sahdev^19^, Allan Hackshaw^4^, Tara D. Barwick ⃰ ^2,3^, Andrea G. Rockall ⃰ ^2,3^

^1^ The Institute of Genetics & Cancer, The University of Edinburgh, Edinburgh, UK

^2^ Department of Radiology, Imperial College Healthcare NHS Trust, London, UK

^3^ Department of Surgery and Cancer, Faculty of Medicine, Imperial College London, London, UK

^4^ Cancer Research UK & UCL Cancer Trials Centre, London, UK

^5^ Department of Cancer Imaging, School of Biomedical Engineering and Imaging Sciences, King’s College London, St. Thomas’ Hospital, Westminster Bridge Rd, London, UK

^6^ Department of Radiology, Royal Marsden Hospital NHS Foundation Trust, London, UK

^7^ Department of Gynaeoncology, Royal Marsden Hospital NHS Foundation Trust, London, UK

^8^ King’s College London and Guy’s and St Thomas’ PET Centre, Guy’s and St Thomas’ NHS Foundation Trust, London, UK

^9^ NIR & MR Physics, Department of Clinical Imaging and Medical Physics, Guys and St Thomas’s NHS Foundation Trust, London, UK

^10^ School of Biomedical Engineering and Imaging Sciences, King’s College London, UK

^11^ Department of Radiology, Beth Israel Deaconess Medical Center, Harvard Medical School, Boston MA, USA

^12^ NIHR Clinical Research Facility, Lancashire Teaching Hospitals NHS Foundation Trust, Preston, UK

^13^ Department of Cellular Pathology, Lancashire Teaching Hospitals NHS Foundation Trust, Preston, UK

^14^ Department of Gynaeoncology, Imperial College Healthcare NHS Trust, London, UK

^15^ Department of Cancer and Genomic Science, University of Birmingham and Pan Birmingham Gynaecological Oncology Centre, Midlands Metropolitan University Hospital, Birmingham, UK

^16^ Wolfson Institute of Population Health, Queen Mary University of London, Charterhouse Square, London, UK

^17^ Department of Gynaecological Oncology, Barts Health NHS Trust, London, UK

^18^ UK Department of Health Services Research, London School of Hygiene & Tropical Medicine, London, UK

^19^ Dept of Radiology, St Bartholomew’s Hospital, Barts Health NHS Trust, London, UK

**Supplementary Materials**

| **Characteristic** | **n = 112** |
| --- | --- |
| Median age (range), years | **65 (25 – 81)** |
| Median BMI (range), kg/m^2^ | **28 (15 – 50)** |
| Primary tumour histology |  |
| Cervical (n = 36) |  |
| Adenocarcinoma | **12** |
| Adenosquamous | **4** |
| Squamous cell | **19** |
| Unknown | **1** |
| Endometrial (n = 76) |  |
| Endometrioid | **42** |
| Serous/clear cell | **31** |
| Other | **2** |
| Unknown | **1** |
| FIGO stage (on MRI) |  |
| Cervical (n = 36) |  |
| 1B1 | **31** |
| 1B2 | **2** |
| 2A1 | **2** |
| 2B | **1** |
| Endometrial (n = 76) |  |
| 1A | **28** |
| 1B | **19** |
| 2 | **9** |
| 3A | **2** |
| 3C | **13** |
| 4B | **5** |

Table 1 - Demographics of patients enrolled onto the MAPPING study and eligible for analysis by meeting the primary reference standard of surgically confirmed nodal histology.


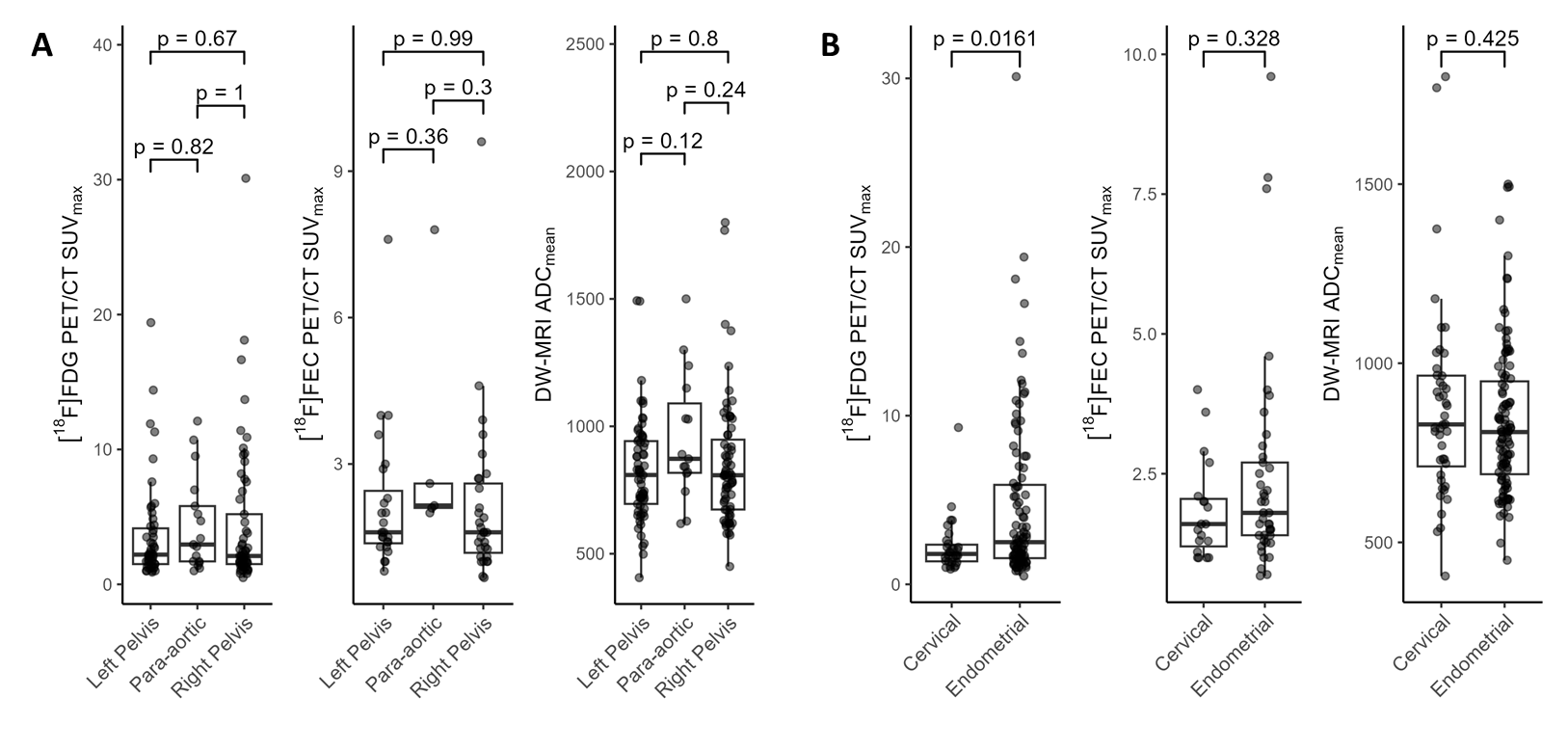


**Fig. 1** (A) Quantitative measures of measured lymph nodes of each region. One-way ANOVA with Tukey’s post-hoc test was used to determine significance between regions. (B) Quantitative measures of measures lymph nodes in endometrial cancer and cervical cancer patients. Mann-Whitney U-test was used to determine significance between endometrial and cervical cancer. [^18^F]FDG PET/CT (left), [^18^F]FEC PET/CT (middle), and DW-MRI (right).


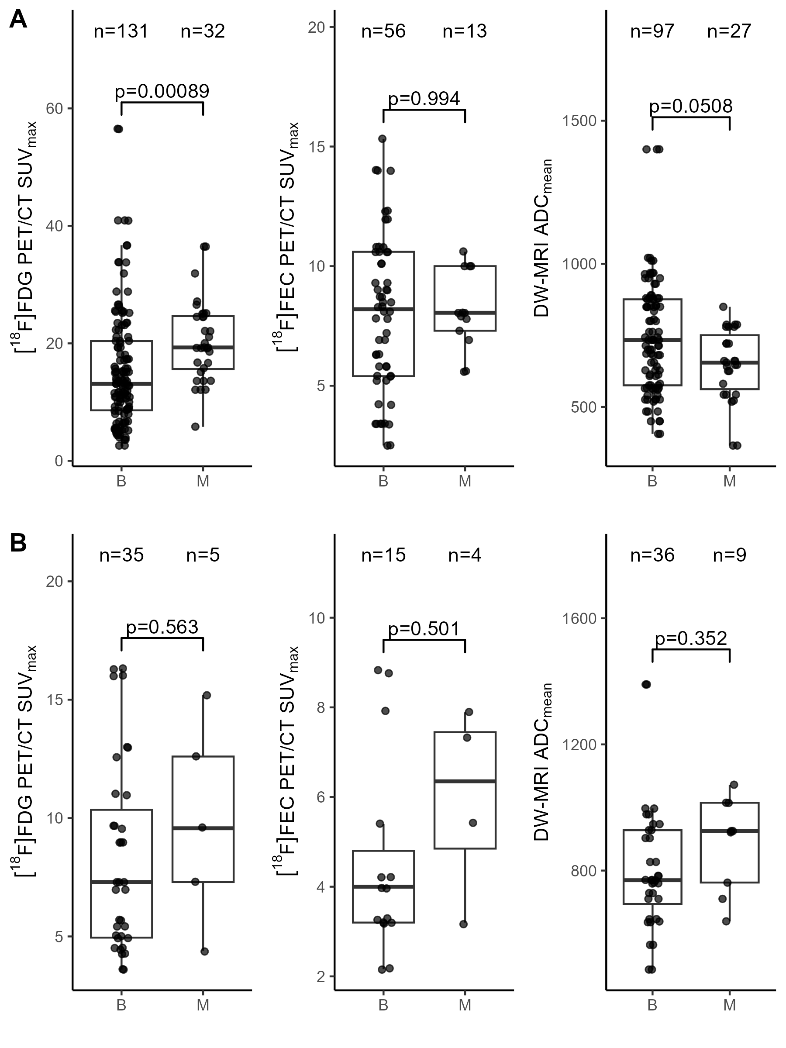


**Fig. 2** (A) SUVmax on FDG-PET/CT was significantly elevated in primary tumours in patients with malignant (M) lymph nodes compared to those with benign (B) lymph nodes in endometrial cancer. (B) No significant difference in quantitative measures of the primary tumour were found for any imaging modality in cervical cancer. Points represent patients; patients are considered to have metastatic disease if they have one or more regions with positive nodal histology.


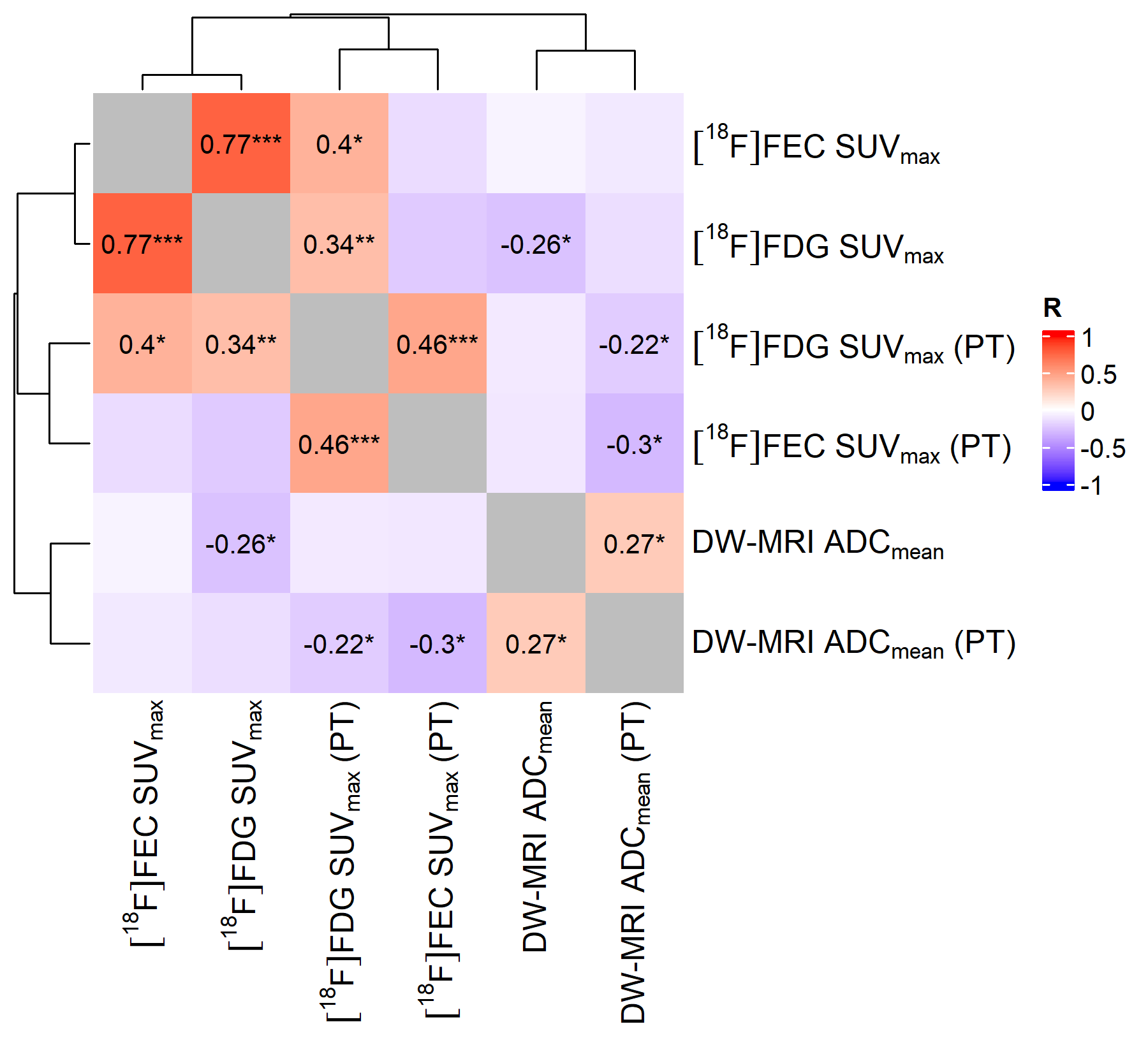


**Fig. 3** Pairwise Pearson correlation coefficients between raw quantitative measures from [^18^F]FDG PET/CT, [^18^F]FEC PET/CT, and DW-MRI in endometrial cancer. PT represents measurements from the primary tumour; the remaining measurements represent nodal regions. ***, p $<$ 0.001, **, p $<$ 0.01, *, p $<$ 0.05.

*
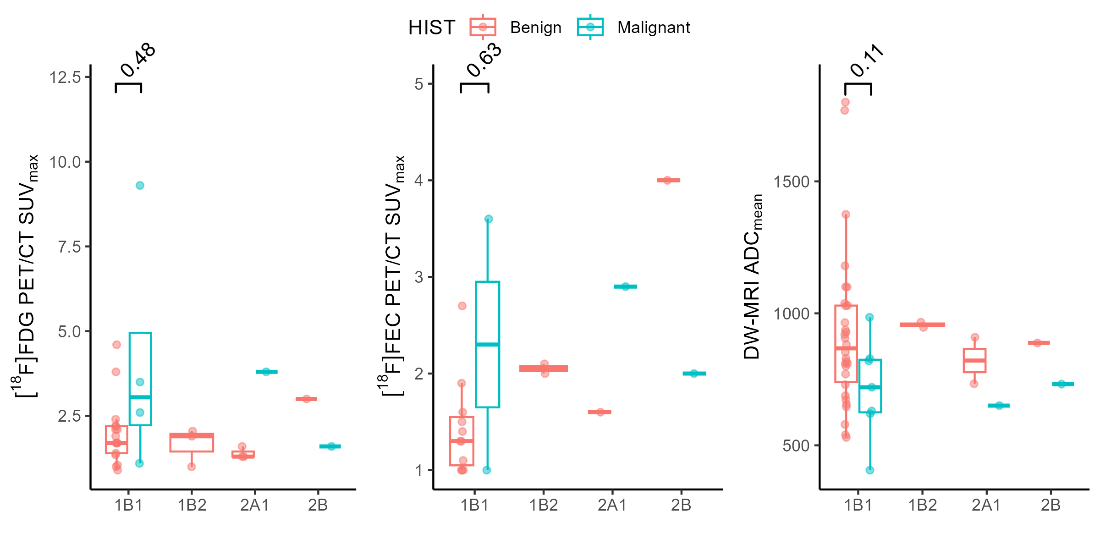

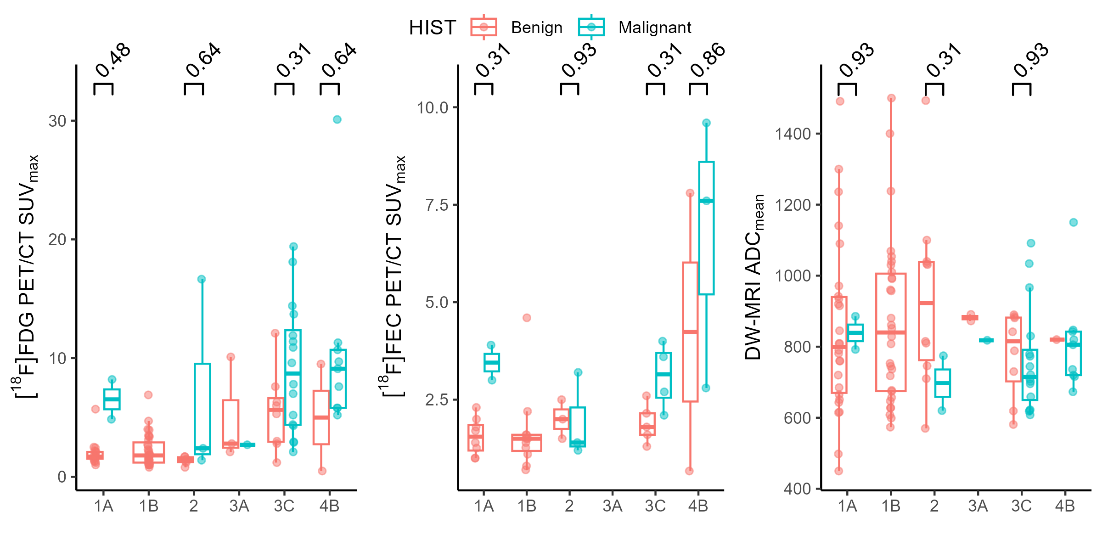
*

***Fig. 4*** *Quantitative measures from DW-MRI, FDG-PET/CT and FEC-PET/CT of benign and malignant lymph nodes association with FIGO stage. Top, cervical cancer; bottom, endometrial cancer.*

*
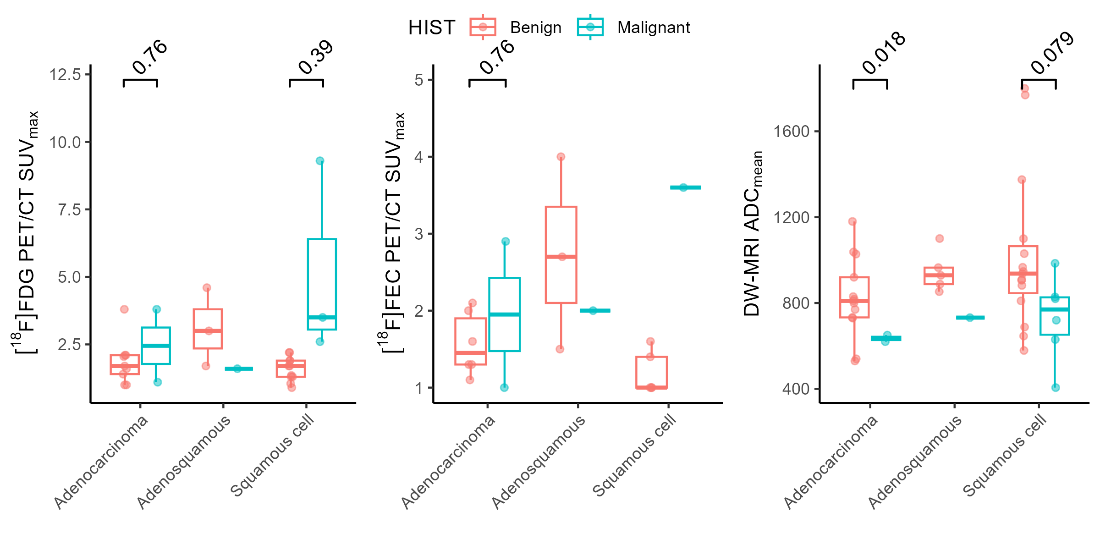

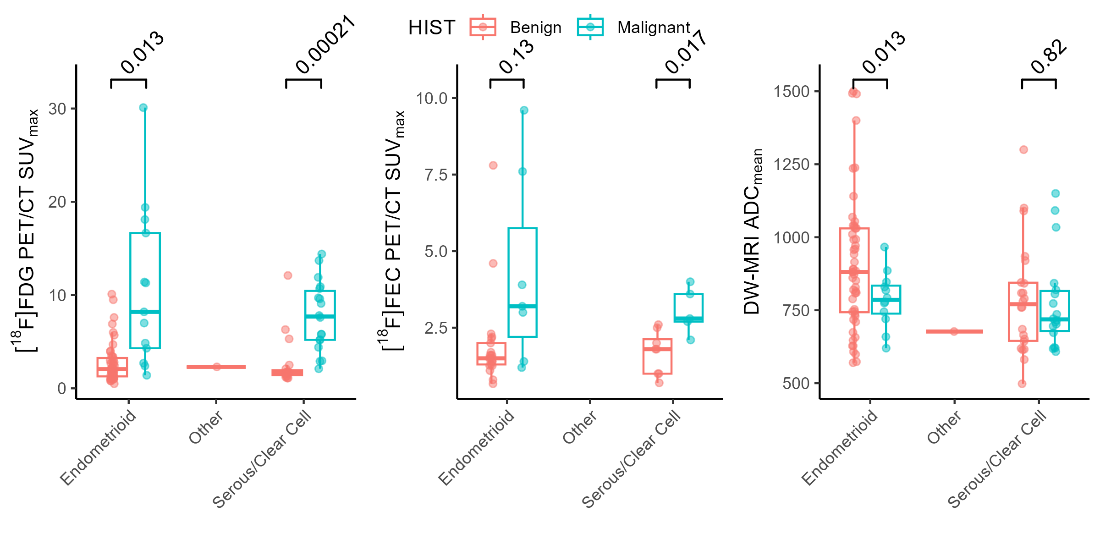
*

***Fig. 5*** *Quantitative measures from DW-MRI, FDG-PET/CT and FEC-PET/CT of benign and malignant lymph nodes by primary tumour histology. Unknown histologies have been removed from the figures. Top, cervical cancer; bottom, endometrial cancer.*

|  |  |  |  | **Quantitative** | | | | | | | | |
| --- | --- | --- | --- | --- | --- | --- | --- | --- | --- | --- | --- | --- |
|  |  | OC | NR | TP | FN | TN | FP | Sensitivity (%) | Specificity (%) | PPV (%) | NPV (%) | F1 (%) |
| [^18^F]FDG PET/CT | SUV_max_ | 2.6 | 30 | 3 | 3 | 21 | 3 | 77.4 (61.5-91.7) | 85.3 (76.1-93.2) | 70.6 (54.5-85.7) | 89.2 (81.0-96.6) | 73.8 (60.0-84.2) |
|  | STAR | 0.004861 | 18 | 3 | 3 | 11 | 1 | 79.3 (63.3-93.5) | 79.2 (65.9-89.8) | 69.7 (51.7-85.3) | 86.4 (75.0-95.7) | 74.2 (59.1-85.3) |
|  | NTR | 0.397074 | 19 | 1 | 3 | 13 | 2 | 53.3 (34.5-70.9) | 80.6 (70.5-90.5) | 57.1 (38.1-76.9) | 78.1 (67.2-87.9) | 55.2 (37.3-70.6) |
| [^18^F]FEC PET/CT | SUV_max_ | 2.9 | 19 | 2 | 2 | 11 | 4 | 66.7 (38.5-90.9) | 93.1 (83.3-100.0) | 80.0 (50.0-100.0) | 87.1 (73.5-96.9) | 72.7 (47.1-90.9) |
|  | STAR | 0.001613 | 11 | 2 | 2 | 3 | 4 | 81.8 (55.6-100.0) | 70.6 (45.0-93.3) | 64.3 (35.7-90.0) | 85.7 (64.3-100.0) | 72.0 (44.4-90.0) |
|  | NTR | 0.367554 | 14 | 2 | 2 | 6 | 4 | 58.3 (30.0-85.7) | 79.2 (61.9-95.2) | 58.3 (30.0-85.7) | 79.2 (62.5-94.7) | 58.3 (31.6-78.6) |
| DW-MRI | ADC_mean_ | 732 | 44 | 4 | 5 | 21 | 14 | 80.0 (63.0-93.1) | 45.5 (34.2-56.4) | 36.4 (25.0-49.2) | 85.4 (72.3-95.1) | 50.0 (37.2-62.0) |
|  | NTR | 1.064865 | 28 | 6 | 1 | 14 | 7 | 88.0 (74.1-100.0) | 21.8 (11.1-32.7) | 33.8 (23.0-45.7) | 80.0 (54.5-100.0) | 48.9 (35.9-60.6) |
|  |  |  |  | **Visual** | | | | | | | | |
|  |  |  | NR | TP | FN | TN | FP | Sensitivity (%) | Specificity (%) | PPV (%) | NPV (%) | F1 (%) |
| [^18^F]FDG PET/CT |  |  | 30 | 2 | 4 | 24 | 0 | 33.3 (0.0-75.0) | 100.0 (NA-NA) | 100.0 (NA-NA) | 85.7 (71.4-96.6) | 50.0 (22.2-85.7) |
|  |  |  | 18 | 2 | 4 | 12 | 0 | 33.3 (0.0-75.0) | 100.0 (NA-NA) | 100.0 (NA-NA) | 75.0 (52.9-94.1) | 50.0 (22.2-88.9) |
|  |  |  | 19 | 1 | 3 | 15 | 0 | 25.0 (0.0-83.0) | 100.0 (NA-NA) | 100.0 (NA-NA) | 83.3 (63.2-100.0) | 40.0 (25.0-100.0) |
| [^18^F]FEC PET/CT |  |  | 19 | 1 | 3 | 15 | 0 | 25.0 (0.0-100.0) | 100.0 (NA-NA) | 100.0 (NA-NA) | 83.3 (64.7-100.0) | 40.0 (25.0-100.0) |
|  |  |  | 11 | 1 | 3 | 7 | 0 | 25.0 (0.0-75.0) | 100.0 (NA-NA) | 100.0 (NA-NA) | 70.0 (40.0-90.9) | 40.0 (25.0-100.0) |
|  |  |  | 14 | 1 | 3 | 10 | 0 | 25.0 (0.0-80.0) | 100.0 (NA-NA) | 100.0 (NA-NA) | 76.9 (53.8-100.0) | 40.0 (25.0-100.0) |
| DW-MRI |  |  | 44 | 2 | 7 | 33 | 2 | 22.2 (0.0-50.0) | 94.3 (85.7-100.0) | 50.0 (0.0-100.0) | 82.5 (70.0-92.7) | 30.8 (13.3-62.5) |
|  |  |  | 28 | 1 | 6 | 19 | 2 | 14.3 (0.0-50.0) | 90.5 (77.3-100.0) | 33.3 (0.0-100.0) | 76.0 (57.7-92.0) | 20.0 (14.3-57.1) |

**Table 2** Diagnostic performance of all quantitative measures and visual assessment in cervical cancer. Visual assessments of nodal regions were matched to quantitative measurements and diagnostic performances estimated with β = 1. Diagnostic performance is shown with 95% confidence intervals in brackets. Asterisks represent FDR-adjusted p-values on McNemar’s test of sensitivity and specificity; *, p < 0.001. OC, optimal cut-off; NR, number of regions; TP, true positive; FN, false negative; TN, true negative; FP, false positive.
